# Differential expression of long non-coding RNAs in three genetic lines of rainbow trout in response to infection with *Flavobacterium psychrophilum*

**DOI:** 10.1038/srep36032

**Published:** 2016-10-27

**Authors:** Bam Paneru, Rafet Al-Tobasei, Yniv Palti, Gregory D. Wiens, Mohamed Salem

**Affiliations:** 1 Department of Biology and Molecular Biosciences Program, Middle Tennessee State University, Murfreesboro, TN, 37132, U.S; 2 Computational Science Program, Middle Tennessee State University, Murfreesboro, TN 37132, U.S; 3 The National Center for Cool and Cold Water Aquaculture, USDA Agricultural Research Service, Kearneysville, WV 25430, U.S

## Abstract

Bacterial cold-water disease caused by *Flavobacterium psychrophilum* is one of the major causes of mortality of salmonids. Three genetic lines of rainbow trout designated as ARS-Fp-R (resistant), ARS-Fp-C (control) and ARS-Fp-S (susceptible) have significant differences in survival rate following *F. psychrophilum* infection. Previous study identified transcriptome differences of immune-relevant protein-coding genes at basal and post infection levels among these genetic lines. Using RNA-Seq approach, we quantified differentially expressed (DE) long non-coding RNAs (lncRNAs) in response to *F. psychrophilum* challenge in these genetic lines. Pairwise comparison between genetic lines and different infection statuses identified 556 DE lncRNAs. A positive correlation existed between the number of the differentially regulated lncRNAs and that of the protein-coding genes. Several lncRNAs showed strong positive and negative expression correlation with their overlapped, neighboring and distant immune related protein-coding genes including complement components, cytokines, chemokines and several signaling molecules involved in immunity. The correlated expressions and genome-wide co-localization suggested that some lncRNAs may be involved in regulating immune-relevant protein-coding genes. This study provides the first evidence of lncRNA-mediated regulation of the anti-bacterial immune response in a commercially important aquaculture species and will likely help developing new genetic markers for rainbow trout disease resistance.

World aquaculture industries suffer considerable economic losses annually because of infectious diseases^1^. *Flavobacterium psychrophilum (Fp)*, a causative agent of Bacterial Cold Water Disease (BCWD), saddleback disease, fry mortality syndrome, or rainbow trout fry syndrome causes significant loss of trout and salmon each year and is a threat to many other salmonids (see review ref. 2). Infection of rainbow trout with *Fp* results in mortality of up to 30% and several complications in the survivors^3^. Originally, the pathogen was considered to be endemic to North America but in recent years it has been reported from almost every continent^4^. Multiple routes of transmission^5^, wide geographical distribution, the ability of pathogen to cope with harsh survival condition^5^, limited chemotherapeutic agents, and lack of a commercial vaccine make control measures inefficient. Live-attenuated *Fp* vaccines can provide protection against BCWD but environmental safety is a concern (see review ref. 6).

Harnessing the host’s immune system by selective breeding is a strategy being pursued to improve farmed fish health^7^. In order to improve resistance of rainbow trout against *Fp*, the National Center for Cool and Cold Water Aquaculture (NCCCWA) started a family-based selective breeding program in 2005. A closed genetic line, designated ARS-Fp-R, has undergone multiple generations of selection for increased survival following standardized challenge. This line has improved disease resistance against *Fp* infection in both laboratory and field settings compared to a susceptible (ARS-Fp-S) and randomly bred control (ARS-Fp-C) lines^8^. Previously, we performed global expression analysis of protein-coding genes in these genetic lines upon *Fp* challenge^9^. The study identified a large number of DE protein-coding genes among genetic lines, a significant proportion of which were genes with described roles in the immune response, especially the innate immune system. We demonstrated transcriptome differences between lines in the absence of infection. However, altered transcriptome abundance of lncRNAs among genetic lines after mock and *Fp* infection was not addressed.

LncRNAs have appeared as critical regulators of transcription and post-transcriptional events of protein-coding genes^10^. LncRNAs regulate diverse cellular processes, including disease, immunity, development and cell proliferation^11^. In mammals, lncRNAs regulate various immune responses including the interferon response, inflammatory processes, and other aspects of innate and adaptive immune responses^12^,^13^,^14^,^15^,^16^,^17^. TLR signaling and inflammatory responses increase the expression of lncRNA-Cox2 that regulates both activation and repression of innate response genes^13^. LncRNA NeST controls susceptibility to Theiler’s virus and Salmonella infection through epigenetic regulation of the interferon-γ locus^17^,^18^. A distinct differential expression profile of lncRNAs in response to microbial infection has been reported in mammals and salmonids, suggesting involvement of a set of lncRNAs in host defense against microbes^11^,^19^. To date, most of the studies in the field of lncRNA influence on immune processes are limited to mammalian species, especially human and mouse. To the best of our knowledge, there are no studies exploring the expression of lncRNAs during host defense against bacterial infection in aquaculture finfish. Such studies are difficult as low evolutionary conservation of lncRNAs across species prevents utilization of the information from mammalian species into aquaculture animals.

The overall objective of this study was to identify lncRNAs that are associated with genetic resistance against *Fp* and to identify immune-relevant protein-coding genes that might be regulated by lncRNAs. To study the expression of lncRNA, we utilized a reference dataset that we recently identified (31,195 lncRNA) in rainbow trout^20^. Using the abovementioned three genetic lines of rainbow trout, we were able to characterize the transcriptome profile of lncRNAs associated with the early response to *Fp* infection. We have identified DE lncRNAs between genetic lines of naive animals and in response to infection, identified their genomic co-localization relative to immune-relevant protein-coding genes, and explored their co-expression relationships to suggest possible regulation of immune-relevant protein-coding genes by lncRNAs.

## Results and Discussion

### Global expression of lncRNA across dataset

Previously, we analyzed mRNA expression in three genetic lines of fish sampled at 1 and 5 days post-*Fp* challenge^9^. In our prior analyses, slightly more than half (51.77%) of the RNA-Seq reads aligned to the 46,585 predicted coding mRNAs and thus considerable sequence information remained unaligned and thus enigmatic. In present study, on average, 8.2% of the total RNA-Seq reads aligned to the 31,195 lncRNAs reference (Supplementary Dataset 1A). 94.5% of the reads were uniquely mapped to the reference. On average, each dataset expressed 87.2% of the putative reference lncRNA’s at RPKM cut off ≥0.5. Out of 31,195 reference lncRNAs, only 933 were not expressed in any dataset (RPKM ≥ 0.5). One possible explanation of the low percentages of aligned read to lncRNA reference compared to protein coding mRNAs might be due to the lower lncRNAs expression compared to mRNAs. Recently, we reported that the average RPKM of the most abundant 40,000 transcripts was 3.49 and 15.69 in LncRNAs and protein-coding genes, respectively^20^. In this study, RNA was sequenced from a whole-body extract, which may be another reason for the low percentage of mappable reads because reference lncRNA dataset was sequenced from about 13 specific tissues. Out of the 933lnc RNAs, only 109 were tissue specific indicating that most of the 933 are very lowly expressed on all tissues.

We utilized pairwise comparisons between different genetic lines and days of infection to identify a sum of 937 DE lncRNA from all comparisons (FDR-P-value < 0.05) (Table 1). Of these, 556 were unique lncRNA showing differential expression in at least one comparison (Supplementary Dataset 2 tab “ALL DEF, non-redundant”). In our previous study using the same genetic lines, ~2,600 DE immune-related and other protein-coding genes were identified in response to *Fp* infection^9^. We quantified the number of DE lncRNAs between different genetic lines and infection statuses (total 24 comparisons) and compared the number with that of DE protein-coding genes. Numbers of DE protein-coding genes and lncRNAs showed moderate positive correlation (R^2^ = 0.40, p = 0.0011) (Table 1). In general, within each pair-wise comparison, fewer differentially regulated lncRNA were identified as compared to DE protein coding transcripts (Table 1). This may, in part, be, due to the overall lower expression level of lncRNA as compared to protein-coding genes^21^. Numbers of the DE protein-coding genes as well as lncRNAs positively correlated with bacterial load in the body. The susceptible line showed more DE lncRNAs as well as protein-coding genes compared to the resistant and control genetic lines (Table 1). Similarly, more transcripts were expressed on day 5 of infection than on day 1. Correlation between body bacterial load and the number of DE lncRNAs on the 5^th^ day of infection in control, susceptible and resistant genetic lines was strongly positively correlated (R^2^ > 0.99); however, correlation of body bacterial load with the number of DE protein coding-genes was moderately positive (R^2^ = 0.34). This finding suggests that, like protein-coding genes, lncRNAs may play a role in the host defense against *Fp.* Expression trends of seven randomly chosen regulated lncRNAs was verified by real time PCR. A consistent trend (R^2^ = 0.84) between RNA-Seq and qPCR was observed, albeit with a somewhat lower relative expression measured by qPCR for 6 of the 7 measured lncRNA’s (Supplementary Dataset 1B). Information about primers and the real time PCR cycling program is provided in Supplementary Dataset 1C.

Recently, we reported tissue specificity of lncRNAs in rainbow trout^20^. A total of 35 DE lncRNAs were selectively expressed in specific tissues, 10 of them were gill-specific. Out of 13 vital tissues, liver, spleen and head kidney did not have any DE lncRNA. Spleen and head kidney lymphoid organ are mainly involved in generation of antibody response and other humoral components of immune system, but in early phase of BCWD, the first line of defense includes skin, alimentary tract lining, and gill^22^.

### Differential expression of lncRNAs between *Fp* infected and PBS injected fish

LncRNAs are involved in the host immune response by regulating various immune-related genes^12^,^13^,^14^,^15^,^16^,^17^. In this study, we initially investigated DE lncRNAs associated with *Fp* injection at days 1 and 5 post-challenge. Pairwise comparison between challenged and time- and line-matched PBS-injected animals identified 327 unique lncRNAs with altered expression (fold change ±2 and FDR-corrected p value < 0.05) (Supplementary Dataset 2 tabs 1–7, and tab “All Fp vs PBS, non-redundant”).

In order to identify lncRNAs that are broadly involved in the response to infection with *Fp*, we quantified the DE lncRNAs (and their correlated protein-coding genes) that were differentially regulated in all three genetic lines upon infection. On the 5^th^ day of infection, 12 lncRNAs were significantly upregulated (>2-fold) in all three genetic lines (FDR-corrected *p*- value < 0.05) (Table 2, top panel). These lncRNAs were most highly upregulated in the susceptible line followed by the control and resistant lines. These finding may indicate that these lncRNAs were either upregulated in response to bacterial load or extent of tissue damage caused by bacterial infection. Surprisingly, none of the lncRNAs was downregulated in all three genetic lines.

Among DE lncRNAs, 6 lncRNAs showed fold changes >100 fold following *Fp* challenge (Table 2, bottom panel). Five out of six lncRNAs, all three upregulated (Omy200018785, Omy200132807 and Omy100037031) and two downregulated (Omy200226560 and Omy100064313), exhibited fold change only in one particular ‘genetic line-by-day of infection’ comparison.

### Relationship between differentially expressed lncRNAs and immune-related protein-coding genes

LncRNAs can be classified as genic or intergenic based on their physical location in genome relative to protein coding gene^20^. Classification of all 556 DE lncRNA is given in Supplementary Dataset 1D. Lack of lncRNAs sequence conservation across species^21^ makes their annotation difficult. In addition, currently there are no enough literature or database resources for rainbow trout and other salmonids to study lncRNAs’ involvement with the host immune system. Therefore, in an effort to implicate association between DE lncRNAs, identified in this study, and the fish defense system, we followed the following criteria based on our prior knowledge of lncRNAs classification and the genetic lines that we used in this study.

#### Differentially expressed lncRNAs that overlap in position and correlate with expression of immune-related protein-coding genes

Several lncRNAs have been identified that regulate expression of neighboring genes acting in *cis* configuration^23^,^24^. Therefore, we searched for DE lncRNAs that were partially or completely overlapping with protein-coding genes in the trout genome. Out of 556 DE lncRNAs, 92 overlapped with protein-coding loci in sense or antisense orientation (Supplementary Dataset 3). Out of the 92 overlapped genes, 36 genes had hits to KEGG pathways, of them 8 different genes were involved in immunity pathways (such as TNF and mTOR signaling pathways) and 4 genes were associated with microbial diseases (such as *Staphylococcus aureus* infection and viral carcinogenesis). There were 3 genes common in both sets of these pathways which are Complement component 5, Signal transducer and activator of transcription 3 and Cyclic AMP-responsive element-binding protein 5.

In order to identify possible relationships between DE lncRNAs and protein-coding genes that physically overlap with them, we compared their expression patterns across 24 different samples that included different genetic lines and infection statuses. Normalized expressions values of the transcripts used to generate clusters are provided in Supplementary Dataset 4. The DE lncRNAs and their overlapping protein-coding genes with a strong expression correlation are listed in Table 3. Overall, we identified 13 protein-coding genes that had strong expression correlation (R^2^ ≥ 0.70) with their overlapping lncRNAs and 6 of those protein-coding genes had already described role in immune system. Consistent with this observation, previous studies suggested overlapped genomic localization of immunity associated lncRNAs with protein coding genes of immune system^25^.

Some lncRNAs showed interesting correlated expression pattern with immune-related protein coding genes post *Fp* challenge and were selected for the following further discussion.

**Figure 1:**
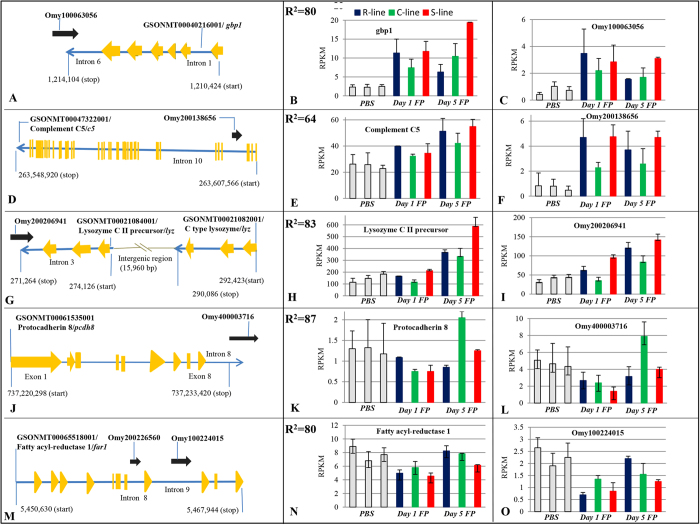
Genomic location of selected differentially expressed lncRNAs relative to protein-coding genes with immune-related functions and their expression patterns among PBS injected and day 1 and day 5 post-*Fp* challenged fish of different genetic lines. Omy100063056 is partially overlapped with intron 6 of *gbp1* in antisense orientation (**A**) and their expression is positively correlated (R^2^ = 0.80) (**B,C**). Omy2001386656 is within intron of complement C5 in antisense orientation (**D**) and they show correlated expression pattern between PBS and *Fp* injected fish (R^2^ = 0.64) (**E,F**). Omy200206941 partially overlaps with intron of lysozyme CII precursor in antisense orientation (**G**) and shows correlated expression pattern with the lysozyme CII precursor (R^2^ = 0.83) (**H,I**). Omy400003716 partially overlaps with intron of protocadherin 8 in sense orientation (**J**) and shows strong positive expression correlation with the protocadherin 8 (R^2^ = 0.87) (**K,L**). Fatty acyl-reductase 1 has one sense lncRNA in each intron 8 (Omy200226560) and 9 (Omy100224015) (**M**) and shows positive expression correlation with both the lncRNAs. Expression pattern of fatty acyl reductase 1 and Omy100224015 is given in figure (**N,O**).

LncRNA Omy100063056 partially overlapped with intron 6 of interferon induced guanylate binding protein-1 like (*gbp1*) (GSONMT00040216001) in antisense orientation and their expression pattern was positively correlated (R^2^ = 0.80) (Fig. 1A–C). RPKM (reads per kilobase per million) count showed that both Omy100063056 and *gbp1* gene transcript were upregulated on day 1 and 5 post-challenge. Upregulation on day 5 was greater in the susceptible line relative to control and resistant lines. *GPB1* gene transcript also shows correlated expression with lncRNA in human^26^. Previous reports suggested that *gbp1* is one of the differentially regulated immune response genes against microbial pathogens in salmon and trout^9^,^27^.

LncRNA Omy200128656 was located in intron 11 of complement C5 (*c5*) (GSONMT00047322001) gene in antisense orientation and their expression was positively correlated (R^2^ = 0.64) (Fig. 1D–F). Expression of *c5* gene transcript was increased by day 5 post-infection and expression of Omy200128656 was upregulated on days 1 and 5 post-challenge. In human, lncRNA C5T1lncRNA, located in 3′UTR of the *C5*, showed upregulated expression upon immune stimulation and its knockdown showed corresponding decrease in transcript level of *C5* mRNA^28^. However, unlike in human, lncRNA Omy200128656 in trout is located in intron 11 of the *c5* gene.

LncRNA Omy200206941 was partially overlapped with intron 4 of lysozyme CII precursor (*lyz*) (GSONMT00021084001) gene in antisense orientation and the expression was positively correlated (R^2^ = 0.83) (Fig. 1G–I). Its expression was also positively correlated with another C type lysozyme (*lyz*) (GSONMT00021082001) gene transcript located about 18 kb away in the same chromosome (R^2^ = 0.88). All these three transcripts showed upregulation on day 5 post-challenge. Consistent with this upregulated expression post challenge, it has been established that C type lysozyme is an important component of innate immune system in salmonid fish^29^. In addition, a neighboring antisense non-coding RNA, LINoCR, is involved in induction of lysozyme locus upon lipopolysaccharide stimulation in chicken^30^.

LncRNA Omy400003716 partially overlapped with intron 8 of protocadherin 8 (*pcdh8*) (GSONMT00061535001) in sense orientation and the expression was highly positively correlated (R^2^ = 0.87) (Fig. 1J–L). RPKM count between PBS and *Fp* challenged fish showed that both Omy400003716 and *pcdh8* gene transcript were downregulated in day 1 post infection relative to naïve and day 5 post-challenged fish.

Two lncRNAs Omy200226560 and Omy100224015 were in intron 8 and 9 of fatty acyl-reductase 1 (*far1*) (GSONMT00065518001) respectively and they positively correlated with the *far1* gene transcript with correlation coefficient (R^2^) of 0.36 and 0.80 respectively (Fig. 1M–O). These three transcripts showed downregulation on day 1, post challenge relative to PBS injected, and day 5, post-*Fp* challenged fish.

Strand orientation of Omy200138656, Omy200206941 and Omy300084989 lncRNAs transcripts were confirmed by strand specific PCR relative to their counterpart protein coding loci (Supplementary Dataset 1E).

#### Differentially expressed lncRNAs that neighbor and correlate with expression of immune-related protein-coding genes

Out of 556 DE lncRNAs, 464 were intergenic without overlap with protein-coding loci in the trout genome. In order to identify the immune-relevant protein-coding genes that were clustered around DE lncRNAs in the genome, we chose protein-coding genes within a 50 kb distance on both sides of DE lncRNAs and performed KEGG pathway analysis of the neighboring protein-coding genes^31^. Out of 464 DE intergenic lncRNAs, 371 had protein-coding genes within 50 kb distance in the genome. A total of 290 genes neighboring to DE lncRNAs had hits to KEGG pathways, of them 51 different genes were related to immunity pathways, 49 genes were involved in microbial infection processes and 28 genes were common in both sets of these pathways (Supplementary Dataset 5).

In the immune system category, most of the KEGG hits were involved in chemokine signaling, platelet activation, complement system, TNF signaling, T cell receptor signaling, Fc gamma R-mediated phagocytosis, Toll-like receptor signaling, phagosome, cytokine-cytokine receptor interaction, NOD-like receptor signaling, leukocyte trans-endothelial migration and others (Supplementary Dataset 5). Similarly, in the microbial pathogenesis category, hits were involved in the pathogenesis of various viral, bacterial and protozoal infections like tuberculosis, influenza A, herpes simplex infection, amoebiasis, bacterial invasion of epithelial cell, and other microbial infections. Interestingly, almost half of the hits to immune system were involved in signal transduction pathways. Among the neighboring protein-coding genes, expression patterns of 9 were highly positively correlated with that of lncRNA (R^2^ ≥ 0.70) (Table 4). About half of the protein-coding genes with high correlation in expression patterns with their neighboring lncRNAs were from components of immune system like suppressor of cytokine signaling 3 (SOCS3), complement factor D, ninjurin-1 and ceramide-1 phosphate transfer protein. Previous studies also indicated that many immune relevant lncRNAs are in 5′ or 3′ close proximity of neighboring protein-coding genes^15^,^25^.

#### Differentially expressed lncRNAs that correlate with expression of immune-related protein-coding genes

LncRNAs have ability to work in *cis* as well as in *trans* configuration^32^,^33^,^34^ and can regulate protein-coding genes that are distant in position on the same or different chromosome. In order to identify possible expression correlation of lncRNAs with such protein coding genes, we performed clustering of DE lncRNAs and protein-coding genes based on their expression pattern across 24 samples. This clustering identified several protein-coding genes with correlated expression with DE lncRNAs that were distantly located in the genome (Table 5). Most of the proteins in these clusters were related to the innate immune system, mainly the complement system, cytokines and chemokines, and receptors and transcription factors of the innate immune system signal transduction pathways. The list included chemokine CK1, NF-kappa B inhibitor alpha, c-c motif chemokine 19, and several proteins of the complements system such as factor B, properdin, component C7 and C4b-binding protein alpha (Table 5).

#### Differentially expressed lncRNAs that correlate with expression of several immune-related protein coding genes

**Figure 2:**
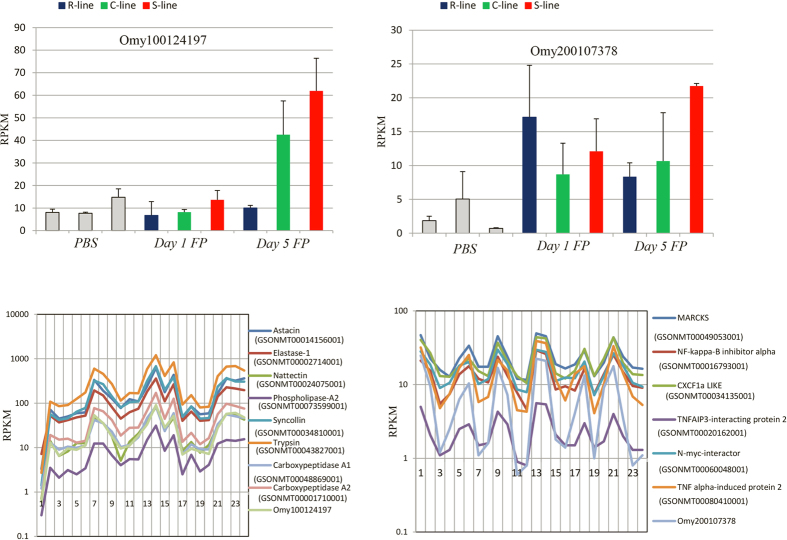
Top two bar graphs show expression patterns of lncRNAs Omy100124197 and Omy200107378 among PBS injected, and day 1 and day 5 post-*Fp* challenged fish in three genetic lines. Respective bottom expression line graphs show expression level of these lncRNAs with different protein-coding genes across 24 samples consisting of different genetic lines and infection statuses. Expression clusters were generated by the Multi-experiment Viewer (MeV) program using a cut off R^2^ minimum of 0.98.

Clustering of DE lncRNAs with protein coding genes based on their expression value identified several protein-coding genes of the immune system correlated with one lncRNA. As an example, lncRNA Omy200107378 was upregulated post *Fp* challenge and its expression was strongly positively correlated with six different protein coding genes, some of which have already established function in immune system (R^2^ > 0.98) (Fig. 2). Similarly, expression of Omy100124197 was strongly correlated with 8 different proteins including matrix metallo-proteinase (Astacin) (GSONMT00014156001), elastase-1 (GSONMT00002714001), nattectin (GSONMT00024075001), phospholipase-A2 (GSONMT00073599001), and syncollin (GSONMT00034810001) (R^2^ > 0.98) (Fig. 2). Role of these correlated proteins in the immune system has already been characterized in different species^35^,^36^,^37^,^38^,^39^,^40^,^41^. Several studies have also reported correlated expression of several immune related protein-coding genes with a single lncRNA^13^.

### LncRNAs expression of naïve fish in different genetic lines

**Figure 3:**
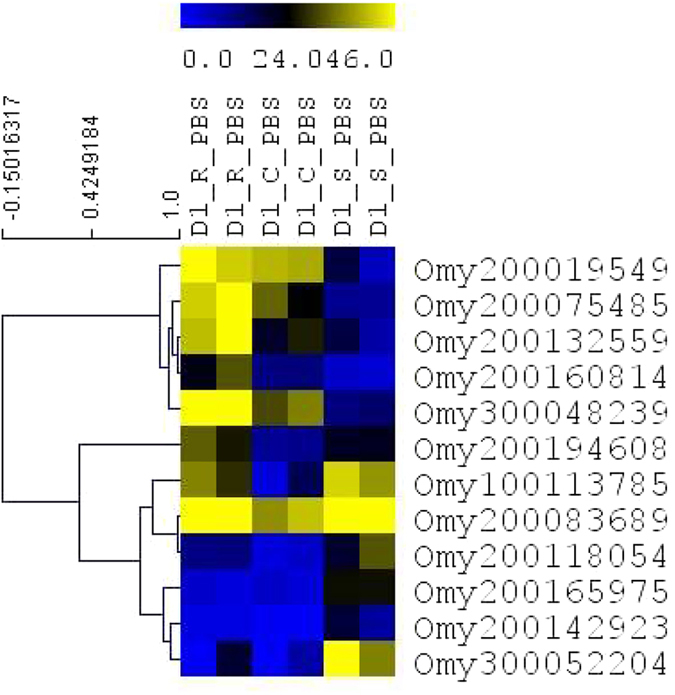
Comparison of transcriptome abundance of selected lncRNAs among naïve fish in all genetic lines. Genes are hierarchically clustered based on their expression pattern. D1 indicates day 1 post challenge and PBS indicates PBS injection. C, R and S represent control, resistant and susceptible genetic lines of the fish.

Three genetic lines of rainbow trout used in this study had significant differences in infection susceptibility to *Fp* as a result of selective breeding^9^. To investigate differences in transcription between lines, we quantified the DE lncRNAs among genetic lines on day 1 following PBS injection. Pairwise comparison identified 32 DE lncRNAs among different genetic lines. Two lncRNAs were DE between the resistant and control lines, 6 lncRNAs between control and susceptible lines, and 24 lncRNAs were DE between resistant and susceptible lines (Supplementary Dataset 2). In our previous study, we identified differences in transcriptome abundance of protein-coding genes among naïve genetic lines^9^. The numbers of DE lncRNAs were smaller but consistent with the numbers of DE protein-coding genes among different naïve genetic lines (Table 1). Expression analysis identified an interesting pattern of transcriptome differences among genetic lines, which correlated with infection susceptibility. LncRNAs Omy200019549, Omy200132559, Omy200160814, Omy200075485 and Omy300048239 were most highly expressed in the resistant line, followed by control and susceptible lines. In contrast, Omy300052204, Omy200142923, Omy200118054 and Omy200165975 were upregulated in the susceptible line relative to the resistant and control lines (Fig. 3). These DE lncRNAs between genetic lines may contribute to differences in infection susceptibility among genetic lines. In consistent with our findings, genetic variation in lncRNAs was shown to be associated with human disease resistance/susceptibility^42^,^43^.

### Difference in transcriptome abundance of lncRNAs among genetic lines after infection

**Figure 4:**
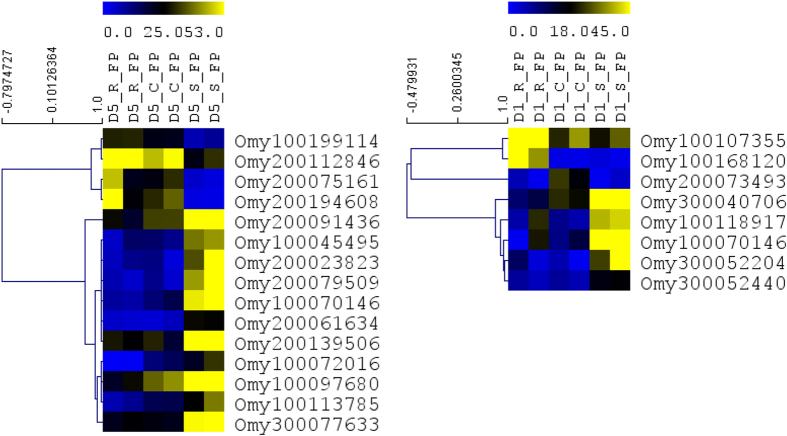
Comparison of transcriptome abundance of selected lncRNAs among genetic lines after infection with *Fp.* Genes are hierarchically clustered based on their expression pattern. D1 and D5 indicate day 1 and day 5 of sampling after injection. *Fp* indicates *Fp* injection. C, R and S represent control, resistant and susceptible genetic lines of the fish.

Induction and activation of adaptive and some of the innate immune components requires pathogen entry into the host suggesting that basal naïve transcriptome level may not be sufficient enough to explain the differences in the ability of the control, susceptible, and resistant fish lines to clear the pathogen. Therefore, we reasoned that, in addition to differences in naïve lncRNA abundance, the genetic lines had altered ability to express immune-relevant transcripts following pathogen challenge. To investigate this point, we quantified DE lncRNAs among genetic lines on days 1 and 5 following *Fp* infection. Pairwise comparison identified 149 DE lncRNAs between genetic lines combined from the 1^st^ and 5^th^ days of infection (Table 1 and Supplementary Dataset 2). On 5^th^ day of infection, there were 83 lncRNAs DE between resistant and susceptible lines; 21 lncRNAs between resistant and control lines, and 5 lncRNAs between control and susceptible lines. On 1^st^ day of infection, these numbers were 15, 12 and 13 respectively (Supplementary Dataset 2). Similarly, on the 1^st^ day of infection majority of the lncRNAs were upregulated on susceptible line relative to two other genetic lines. The expression number of DE’s correlated with the gradient of bacterial load between the three genetic lines: S > C > R. Previous report also indicated correlation of lncRNAs expression with microbial load^44^. Figure 4 shows abundance of selected hierarchically clustered lncRNAs among genetic lines after infection with *Fp*. On the 5^th^ day of infection, most of the lncRNAs were upregulated in the susceptible line compared to control and resistant lines, with only Omy200112846, Omy200075161, Omy200194608 and Omy100199114 exhibiting opposite trend in expression level (Fig. 4).

### LncRNA transcriptome change as the disease progress from day 1 to day 5

**Figure 5:**
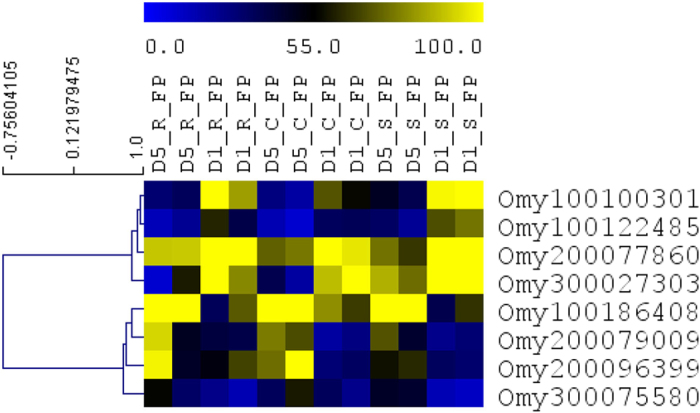
Comparison of transcriptome abundance of selected lncRNAs between day 1 and day 5 of *Fp* injection in each genetic line. Genes were hierarchically clustered based on their expression pattern. D1 and D5 indicate day 1 and day 5 of sampling after injection and Fp indicates *Fp* injection. C, R and S represent control, resistant and susceptible genetic lines of the fish.

During the course of infection, the host can utilize different immune components at different stages of disease, which requires change in expression of immune-relevant genes. We reasoned that if lncRNAs regulate the immune system, their transcriptome changes, like that of protein-coding genes, would change as the disease progresses. Pairwise comparison between day 1 and day 5 post-*Fp* challenge identified 137 lncRNAs whose expression was significantly changed during two time points (Supplementary Dataset 2). This finding is consistent with previous report demonstrating change in the number of differentially regulated lncRNAs at different ISAV infection time points in Atlantic salmon^19^. Figure 5 shows abundance of selected hierarchically clustered lncRNAs between day 1 and day 5 of *Fp* injection in each genetic line. As expected, some of the lncRNAs that showed altered expression between day 1 and day 5 post-challenges had strong expression correlation with immune relevant protein coding genes. LncRNAs Omy200174653 had altered expression on day 5 relative to day 1 post challenge in susceptible lines and a strong positive correlation with complement factor D (Table 4). Similarly, Omy100066751 and Omy200107535 exhibited a strong positive expression correlation with tumor necrosis factor alpha-induced protein 2 (tnfaip2) and nuclear factor of kappa light polypeptide gene enhancer in B-cells 2 (NFKB2) (R^2^ = 0.92), respectively (Table 5). NFKB2 is a transcription factor required to maintain normal level of antigen specific antibody production in response to antigen challenge^45^. It is noteworthy that Omy200107535 was one of the 12 lncRNAs that were upregulated on day 5 post challenge relative to naïve fish in all three genetic lines (Table 2). This change in expression pattern of lncRNAs during the course of infection suggests that these lncRNAs may play a role in adjustment of immunity depending on severity and stage of the disease. In addition, these DE lncRNAs might play a role in host pathogen interaction or pathogen life cycle during the course of infection as suggested in previous studies^46^.

### Sequence homology with lncRNAs in Atlantic salmon

Recently differentially regulated lncRNAs in response to infectious salmon anemia virus (ISAV) has been characterized in Atlantic salmon^19^. Out of 556 DE lncRNA in trout genetic lines in various comparisons, 23 showed significant sequence homology with Atlantic salmon lncRNAs that were associated with ISAV infection (query cover > 50%, sequence identity > 90% and E value < 1e-10) (Supplementary dataset 6). Interestingly, out of 23 conserved lncRNA, 17 showed regulated expression in *Fp* injected fish relative to PBS injected naïve animals; and remaining 6 were differently regulated between genetic lines and time points of infection comparison (Supplementary dataset 2). It is worth mentioning that one of the conserved lncRNA, Omy300043066 had strong positive expression correlation with properdin and complement factor b like protein in trout (Table 5) and was one of the 12 lncRNAs that were upregulated during infection in all three genetic lines relative to their PBS injected fish (Table 2). All of the 23-conserved lncRNA were regulated in salmon in response to ISAV, indicating potential role in general immunity rather than being bacterial or virus specific.

### Novel lncRNAs in resistant and susceptible genetic lines

Novel lncRNAs were detected in each genetic line separately by running sequence reads through our previously described lncRNA discovery pipeline^20^. 589 susceptible-specific and 631 resistant-specific novel lncRNAs were predicted. FASTA files are available at http://www.animalgenome.org/repository/pub/MTSU2015.1014/. Correlation analyses of gene expression showed only 9 lncRNAs in moderate correlation (R^2^ ≥ 0.70) with protein coding genes. However, none of these proteins was overlapped with lncRNA or had previously described role in immune system (Supplementary dataset 7). While identification of these lncRNAs were limited to each genetic line, their multiple group ANOVA analysis of gene expression (genetic line X infection status X time point) showed a complex expression pattern (Supplementary dataset7-PCA). Interestingly, two lncRNA (dis_R_00048342 and dis_R_00050098) showed resistant-line specific gene expression regardless of the infection status or the time points. Similarly, three lncRNA (dis_S_00030301, dis_S_00043616 and dis_S_00083595) were susceptible-line specific (Supplementary dataset 7). On the other hand, 20 lncRNAs showed explicit expression after *Fp* infection, regardless of the time of infection or the genetic line. In addition, three lncRNA showed explicit expression between day 1 and day 5 of infection (Supplementary dataset 7). These finding may suggests that genomic selection for BCWD over three generations may have introduced novel genomic variations or genomic reorganization of some lncRNA loci and altered expressions of lncRNAs.

## Conclusion

Thus far, studies on host response to microbial infection in salmonids have given significant attention to changes in protein-coding gene expression. However, lncRNAs have emerged as key regulators of host defense against a wide variety of pathological processes including microbial infection^11^,^12^,^13^,^14^,^15^,^16^,^17^,^19^. Manipulation of individual lncRNAs is sufficient to change the expression of hundreds of immune response genes^13^, and variation in expression of other lncRNA’s alter host susceptibility to different microbial pathogens^17^. In the present study, we quantified DE lncRNAs in response to *Fp* infection, which is an important cause of morbidity and mortality in salmon and trout^2^. This study is novel as we characterized the expression signature of lncRNAs on a genome-wide scale in response to one of the major bacterial infection of a salmonid fish. To our knowledge, regulation of lncRNA during bacterial pathogen challenge has not previously been studied in any aquaculture/fish species.

Using transcriptome-wide datasets of protein-coding genes and lncRNAs across 24 samples, we were able to identify potential immune-relevant and other protein-coding genes correlating with DE lncRNAs. This study identified correlation between the genomic physical proximity and coordinated expression of a large number of immune related and other protein coding genes with that of lncRNAs during BCWD in rainbow trout. In this study, most of the DE lncRNAs (sense and antisense) had significant positive expression correlation (R^2^ > 0.70) with their overlapped and/or neighboring protein coding genes. These results are consistent with human ENCODE project results that showed particularly striking positive correlation of lncRNAs with the expression of antisense coding genes^21^. In trans-acting lncRNAs, the ENCODE project observed that lncRNAs are more positively than negatively correlated with protein-coding genes, a finding consistent with our observation of more frequent positive than negative correlation with distantly located protein coding genes. The positive correlation between lncRNA and protein coding genes suggest potential for co-expression^10^.

This study has characterized DE lncRNAs in response to an initial phase of BCWD (day 1 and 5 post-challenge) and has explored expression correlation of lncRNAs with immune relevant protein coding gene that may play crucial role in pathogenesis or immunity during the early phase of the disease in rainbow trout. Further mechanistic study of the underlying biological relationship between correlated DE lncRNAs and proteins of innate immune system will help understand regulation of pathogenesis/immunity at this crucial phase of infection in juvenile rainbow trout.

## Materials and Methods

### Ethics statement

Fish were maintained at the NCCCWA and all experimental protocols and animal procedures were approved and carried out in accordance with the guidelines of NCCCWA Institutional Animal Care and Use Committee Protocols #053 and #076.

### Experimental animals and RNA-Seq experimental design

Three rainbow trout genetic lines ARS-Fp-R, ARS-Fp-C, and ARS-Fp-S used in this study were developed at National Center for Cool and Cold Water Aquaculture (NCCCWA) rainbow trout breeding program. These genetic lines differ significantly to their susceptibility to *Fp* infection as a result of genetic selection^8^ and we have previously reported the challenge experiment utilized in this study^9^. Briefly, fifty randomly selected fish from each genetic line were assigned to four challenge tanks (total 12 tanks for three genetic lines). At the time of challenge, average body weight was 1.1 g and fish age was 49 days post-hatch. For each genetic line, fish in two tanks were injected with *Fp* (experimental group) and fish in the other two tanks were injected with PBS (control group). Fish were injection challenged with either 4.2 × 10^6^ CFU *Fp* suspended in 10 μl of chilled PBS or PBS alone, and survival was monitored daily for 21 days^9^. For RNA extraction, five individuals were sampled from each tank on days 1 and 5 post infections. Survival at 21 days post-challenge injection was monitored during the experiment. Post-challenge bacterial load in the body was measured in a subset of fish by qPCR and was expressed in terms of *Fp* genome equivalents (GE).

### RNA extraction, library preparation, and sequencing

Tissue sampling, RNA extraction, library preparation and sequencing were done as described previously^9^. Briefly, total RNA was extracted and equal amounts of RNA from five fish were pooled from each of the 12 tanks at each of the two time points (total of 24 pools, n = 120 fish total). cDNA libraries were prepared using Illumina’s TruSeq Stranded mRNA Sample Prep kit following the manufacturer’s instructions. The 24 indexed and barcoded libraries were randomly divided into three groups (eight libraries per group) and sequenced in three lanes of an Illumina HiSeq 2000 (single-end, 100 bp read length). RNA-Seq reads are available at the NCBI Short Read Archive (BioProject ID PRJNA259860, accession number SRP047070).

### Differential gene expression analysis of lncRNAs

Complete description of lncRNA reference dataset with their discovery pipeline has been recently described^20^. From this discovery datasets, a stringently selected set of lncRNAs (31,195) were used as a reference for gene expression analysis. For differential gene expression analysis, sequencing reads from each library were mapped to the lncRNA reference using a CLC genomics workbench. Mapping conditions were, mismatch cost = 2, insertion/deletion cost = 3, minimum length fraction = 0.9 and similarity fraction = 0.9. The expression value of lncRNAs was calculated in terms of RPKM (reads per kilobase per million). EDGE (extraction and analysis of differential gene expression) tests were performed to identify DE genes between various groups, e.g. infected vs. non-infected, day 1 vs. day 5, and one genetic line vs. other with or without *Fp* injection^47^. To control false discovery due to multiple testing, p-values were FDR-corrected. LncRNA was considered significant at a fold-change cutoff value of ±2 and a corrected p-value of less than 0.05.

### Validation of RNA-Seq data by qPCR

From DE lncRNAs in the RNA-Seq study, 7 were randomly selected from the DE day 5 susceptible line for experimental validation using individual (unpooled) samples. RNA isolation, cDNA synthesis and primer design were completed using the same technique as described previously^9^. Briefly, RNAs were treated with Optimize™ DNAase I (Fisher Bio Reagents, Hudson, NH) to eliminate genomic DNA. One microgram of the purified RNA was converted to cDNA using the Verso cDNA Synthesis Kit (Thermo Scientific, Hudson, NH) according to the manufacturer protocol. Reverse transcription was performed using My Cycler™ Thermal Cycler (Bio Rad, Hercules, CA) at 42 °C for 30 min (one cycle amplification) followed by 95 °C for 2 min (inactivation). Blend of random hexamer and oligo (dT) primer (3:1 V/V), at a final concentration of 25 ng/μL, was used to prime the reverse transcription reaction.

The Bio-Rad CFX96™ Real Time System (Bio-Rad, Hercules, CA) in conjunction with SsoAdvanced™ Universal SYBR® Green Supermix (Bio-Rad, Hercules, CA, USA) was used to quantify the amount of the expressed gene of interest in PBS and *Fp* injected whole-body fish homogenates. Each primer was used at a concentration of 0.1 nM/μL and cDNA template was used at a concentration of 0.006 μg/μL. Cycling temperatures were set up according to the manufacturer’s protocol and different annealing temperatures were used depending on primers. Fold change in gene expression was calculated as described previously^9^. Briefly, β-actin (Accession: AJ438158) was used as endogenous reference to normalize each target lncRNA. qPCR data were quantified using delta delta Ct (ΔΔCt) method^48^. Ct-values of β-actin were subtracted from Ct-values of the target gene to calculate the normalized value (ΔCt) of the target lncRNA in both the calibrator samples (PBS-injected) and test samples (*Fp*-injected). The ΔCt value of the calibrator sample was subtracted from the ΔCt value of the test sample to get the ΔΔCt value. Fold change in gene expression in the test sample relative to the calibrator sample was calculated by the formula 2^−ΔΔCt^ and the normalized target Ct values in each infected and non-infected group was averaged. Correlation between gene expression fold-change measured by qPCR and RNA-Seq was performed by Pearson correlation. All statistics were performed with a significance of *P* < 0.05.

### Gene clustering and gene expression correlation

Sequencing reads from all 24 libraries (samples) were mapped to a combined reference sequence consisting of all lncRNAs, that we previously identified^20^, and mRNAs that were identified in the rainbow trout genome^49^. Expression of lncRNAs and protein-coding genes was measured in terms of RPKM. The expression value of each transcript in each sample was normalized using the scaling method^50^. Mean was chosen as normalization value and median mean was chosen as reference. Five percent of the data on both sides of the tail were trimmed. Normalized expression values of transcripts in each sample were used to cluster protein-coding genes and lncRNAs using algorithms in Multi-experiment Viewer (MeV). Clusters were generated with a minimum correlation coefficient of 0.92. During clustering, 30% of the sequences with flat expression values over samples were excluded from cluster generation to prevent uninteresting cluster generation. Correlation in expression of lncRNAs and neighboring/overlapped protein-coding genes was performed in Excel using regression analysis using normalized expressions values of the transcripts.

### Discovery of novel lncRNAs in resistant and susceptible genetic lines

Novel lncRNA were identified according to Al-Tobasei *et al.*^20^. Briefly, sequencing reads from each genetic lines (resistance, control and susceptible) were aligned to a rainbow trout reference genome using TopHat^49^. Cufflinks, Cufflinks compare and Cufflinks Merge were used to predict transcripts in each genetic line. Transcripts shorter than 200 nt were filtered out using in house perl script. Transcripts which had open reading frame (ORF) longer than 100 amino acids were removed. In addition, if ORF of the transcript is longer than 35% of the transcript length, the transcript was filtered out even if the ORF is shorter than 100 amino acids. Subsequently, transcripts were searched against NR protein database (updated on May 2016) using BLASTx, and any transcripts with sequence homology to existing proteins were removed. To remove any remaining protein coding transcripts, coding potential calculator (CPC) was applied to the transcripts (Index value < −1.0). Other classes of non-coding RNAs (e.g. rRNA, tRNA, snoRNA, miRNA, siRNA and others) in the dataset were removed by blasting (BLASTn) the transcripts against multiple RNA databases including genomic tRNA database. Finally, any single exon transcripts within 500 nts of protein coding gene was removed. After these filtration steps, remaining transcripts were considered as putative lncRNAs. To identify lncRNAs specific to a particular genetic line, lncRNAs from one genetic line were compared with lncRNAs from other two genetic lines. Resistant and susceptible specific lncRNA were reported.

## Additional Information

**How to cite this article**: Paneru, B. *et al.* Differential expression of long non-coding RNAs in three genetic lines of rainbow trout in response to infection with *Flavobacterium psychrophilum. Sci. Rep.* **6**, 36032; doi: 10.1038/srep36032 (2016).

**Publisher’s note:** Springer Nature remains neutral with regard to jurisdictional claims in published maps and institutional affiliations.

## Supplementary Material

Supplementary Dataset 1

Supplementary Dataset 2

Supplementary Dataset 3

Supplementary Dataset 4

Supplementary Dataset 5

Supplementary Dataset 6

Supplementary Dataset 7

## Figures and Tables

**Table 1:** Comparison of differentially expressed lncRNA and protein coding genes in response to *Fp* infection.

Comparison	Day, genetic line and infection status	No. differentially expressed protein-coding genes^a^	No. differentially expressed lncRNAs
Infected vs PBS	Day 1 R-line (Fp) vs. R-line (PBS)	515	57
	Day 5 R-line (Fp) vs. R-line (PBS)	428	36
	Day 1 C-line (Fp) vs. C-line (PBS)	20	0
	Day 5 C-line (Fp) vs. C-line (PBS)	2201	54
	Day 1 S-line (Fp) vs. S-line (PBS)	1663	125
	Day 5 S-line (Fp) vs. S-line (PBS)	2225	196
Genetic lines (PBS)	Day 1 R-line (PBS) vs. S-line (PBS)	76	24
	Day 1 R-line (PBS) vs. C-line (PBS)	3	2
	Day 1 S-line (PBS) vs. C-line (PBS)	28	6
	Day 5 R-line (PBS) vs. S-line (PBS)	45	22
	Day 5 R-line (PBS) vs. C-line (PBS)	246	28
	Day 5 S-line (PBS) vs. C-line (PBS)	61	25
Genetic lines (Fp)	Day 1 R-line (Fp) vs. S-line (Fp)	150	15
	Day 5 R-line (Fp) vs. S-line (Fp)	1016	83
	Day 1 R-line (Fp) vs. C-line (Fp)	28	12
	Day 5 R-line (Fp) vs. C-line (Fp)	159	21
	Day 1 S-line (Fp) vs. C-line (Fp)	37	13
	Day 5 S-line (Fp) vs. C-line (Fp)	1758	5
Time points	Day 5 vs. Day 1 R-line (PBS)	1286	26
	Day 5 vs. Day 1 C-line (PBS)	294	36
	Day 5 vs. Day 1 S-line (PBS)	376	14
	Day 5 vs. Day 1 R-line (Fp)	334	22
	Day 5 vs. Day 1 C-line (Fp)	2469	70
	Day 5 vs. Day 1 S-line (Fp)	2434	45

Four different comparisons were made to quantify the differentially expressed transcripts: infected vs. non-infected, one genetic line vs. another without infection, one genetic line vs. another post infection, and day 1 vs. day 5 of infection. Differential expression was considered at fold change ±2 and FDR-corrected p < 0.05. Number of differentially expressed protein coding genes and lncRNAs showed positive correlation (R^2^ = 0.40, *P* = 0.0011).

^a^Data are from^9^.

**Table 2:** 

LncRNAs upregulated in all three genetic lines (>2 fold) upon infection and their expression correlation with protein coding genes							
LncRNA feature ID	Resistant line		Control line		Susceptible line		Correlation with (R^2^)
	EDGE test: Fold change	FDR p-value correction	EDGE test: Fold change	FDR p-value correction	EDGE test: Fold change	FDR p-value correction	
Omy200117486	24.36	0	41.6	0	91.18	0	Interferon-induced guanylate-binding protein 1 (0.82)
Omy100128008	14.95	0	22.23	0	46.4	0	Complement protein component C7-1 (c7-1) (0.82)
Omy200138656	24.3	0.000001	11.63	0.011489	28.75	0	Complement C5 (0.66)
Omy100149048	7.93	0.000004	5.95	0.048727	14.15	0	Unknown
Omy200107378	6.38	0.000062	11.22	0.004248	11.22	0	Nuclear factor of kappa light polypeptide gene enhancer in B-cells 2 (0.92)
Omy200165911	3.68	0.049287	4.5	0.040404	9.98	0	Unknown
Omy100052789	5.51	0.000564	5.13	0.047151	8.65	0	Unknown
Omy200107535	3.71	0.000147	6.16	0.012707	8.12	0	Nuclear factor of kappa light polypeptide gene enhancer in B-cells 2 (0.92)
Omy300025398	4.37	0.006514	5.15	0.002475	4.86	0	Unknown
Omy300085997	3.68	0.000345	3.16	0.043867	4.02	0.001759	Unknown
Omy200206941	3.16	0.000344	2.33	0.015415	3.44	0.000002	Lysozyme C II precursor (0.83)
Omy300043066	3	0.000121	3.74	0.006748	3.03	0.000014	Properdin (0.82) and complement factor b-like (0.89)
**LncRNAs showing drastic (>100) fold change upon infection in one particular genetic line and associated gene in genome**							
**Feature ID**	**EDGE test: Fold change**	**FDR p-value correction**	**Comparison**	**Classification of LncRNA**	**Associated Gene(s) (R**^**2**^)		
Upregulated upon infection							
Omy200018785	136.06	0.001233	D1_S_FP vs D1_S_PBS	Intergenic			
Omy200132807	121.83	0.000167	D5_S_FP vs D5_S_PBS	Intergenic			
Omy100037031	105.28	0.012820	D5_C_FP vs D5_C_PBS	Intergenic			
Downregulated upon infection							
Omy200194608	−168.77	0.000001	D1_S_FP vs D1_S_PBS	Genic, antisense	GSONMG00062425001 si:ch73- protein (0.27)		
Omy200226560	−121.90	0.001972	D1_R_Fp vs D1_R_PBS	Genic, antisense	GSONMG00065518001 (fatty-acyl reductase-1) (0.36)		
Omy100064313	−108.56	0.001716	D1_R_Fp vs D1_R_PBS	Intergenic			

LncRNAs upregulated in all three genetic lines (>2 fold) on 5^th^ day post *Fp* challenge and their expression correlation with protein coding genes (top). LncRNAs showing highest fold change (>100-fold) upon *Fp* infection in at least one genetic line relative to the two other genetic lines and their associated protein coding gene in genome (bottom). Fold change was considered significant if FDR-corrected p value was less than 0.05.

**Table 3:** Correlation between expression patterns of lncRNAs and their overlapping protein-coding genes (R^2^ > 0.70).

LncRNA	Size	Neighboring protein-coding genes (ID)	Overlap	Direction relative to LncRNA	Expression correlation type (R^2^)	Annotation of coding gene	Reference to immunity or pathogenesis function
**Protein-coding genes with known immune function or association with microbial infection**							
Omy100063056	1,263	GSONMT00040216001	Intronic	Antisense	Positive (0.73)	Interferon-induced guanylate-binding protein 1-like	51,52
Omy200083892	1,294	GSONMT00050654001	Intronic	Antisense	Positive (0.84)	Tumor necrosis factor receptor superfamily member 9-like (Tnfrsf9)	9
Omy200080884	1,512	GSONMT00034829001	Exonic	Antisense	Positive (0.93)	Response gene to complement 32 protein (rgc32)	53
Omy200206941	537	GSONMT00021084001	Intronic	Unknown	Positive (0.83)	Lysozyme C II precursor	29
Omy200107012	885	GSONMT00019341001	Intronic	Unknown	Positive (0.89)	Stromal interaction molecule 2-like	54
Omy100228715	297	GSONMT00079494001	Exonic	Unknown	Positive (0.83)	Unnamed protein product/transcobalamin-1 like	55
**Protein-coding genes with no previously described immunity function**							
Omy400008156	668	GSONMT00041383001	Intronic	Unknown	Positive (0.87)	Reticulon-2 like	
Omy300038945	596	GSONMT00049537001	Intronic	Antisense	Positive (0.81)	Cytochrome P450 7B1	
Omy400006181	248	GSONMT00049631001	Intronic	Unknown	Positive (0.77)	Collagen alpha-1(IX) chain-like	
Omy400003716	725	GSONMT00061535001	Intronic	Sense	Positive (0.87)	Protocadherin 8 (pcdh8)	
Omy100224015	683	GSONMT00065518001	Intronic	Antisense	Positive (0.71)	Fatty acyl-CoA reductase 1 (facr1)	
Omy200181316	604	GSONMT00071779001	Exonic	Unknown	Positive (0.82)	Muscular LMNA-interacting protein	
Omy100171980	1,292	GSONMT00073108001	Exonic	Unknown	Positive (0.82)	Immunoglobulin-like and fibronectin type III domain-containing protein 1	

References are provided for protein-coding genes with previously described functions in immunity or association with microbial infection/pathogenesis.

**Table 4:** Correlation between expression patterns of lncRNAs and their intergenic neighboring protein-coding genes (within <50 kb and R^2^ > 0.70).

LncRNA	Size	Neighboring protein-coding genes (ID)	Distance from LncRNA (KB)	Direction relative to LncRNA		Expression correlation type (R^2^)	Annotation of coding gene	Reference to immune or pathogenesis function
**Protein-coding genes with known immune function or association with microbial infection**								
Omy200174653	519	GSONMT00031633001	5.0	Unknown/Intergenic	Positive (0.92)		Complement factor D-like	56
Omy300084989	596	GSONMT00013116001	2.6	Antisense/Intergenic	Positive (0.71)		Suppressor of cytokine signaling	57
Omy300074800	493	GSONMT00003195001	1.1	Unknown/Intergenic	Positive (0.79)		Ninjurin-1	58
Omy200206941	537	GSONMT00021082001	18.6	Unknown/Intergenic	Positive (0.88)		C type lysozyme	29
Omy200073559	2,093	GSONMT00017721001	3.5	Unknown/Intergenic	Positive (0.77)		Ceramide-1-phosphate transfer protein-like	59
**Protein-coding genes with no previously described immunity function**								
Omy200061208	1057	GSONMT00041695001	0.3	Unknown/Intergenic		Positive (0.90)	Coiled-coil transcriptional coactivator b	
Omy200112536	1,059	GSONMT00001821001	18.2	Unknown/Intergenic		Positive (0.88)	Serum albumin 1	
Omy300087476	619	GSONMT00010387001	0.9	Unknown/Intergenic		Positive (0.83)	Neutral amino acid transporter B(0)	
Omy200075445	745	GSONMT00008107001	1.3	Unknown/Intergenic		Positive (0.70)	Hepatocyte nuclear factor 4-beta-like	

References are provided for some of the protein-coding genes with previously described functions in immunity or association with microbial infection/pathogenesis.

**Table 5:** Correlation between expression patterns of lncRNAs and some distantly located (>50 kb or different chromosome) immune-relevant protein-coding genes.

LncRNA	Size	Protein-coding genes (ID)	Expression correlation type (R^2^)	Annotation of coding gene	Reference to immunity or pathogenesis function
Omy100104455	587	GSONMT00024124001	Positive (0.96)	Chemokine CK1	60
Omy200174653	357	GSONMT00051250001	Positive (0.92)	C-C motif chemokine 19 precursor	61
Omy300084989	596	GSONMT00062775001	Positive (0.83)	C4b-binding protein alpha chain precursor	62
Omy300041057	448	GSONMT00042009001	Positive (0.80)	Caspase-8	63
Omy300043066	715	GSONMT00001792001	Positive (0.82)	Properdin	64
Omy300043066	715	GSONMT00027840001	Positive (0.89)	Complement factor b-like	65
Omy200100893	357	GSONMT00051250001	Positive (0.92)	C-C motif chemokine 19 precursor	61
Omy200107378	522	GSONMT00016681001	Positive (0.92)	Nuclear factor of kappa light polypeptide gene enhancer in B-cells 2	45
Omy200107535	948	GSONMT00016681001	Positive (0.92)	Nuclear factor of kappa light polypeptide gene enhancer in B-cells 2	45
Omy200117486	529	GSONMT00005714001	Positive (0.82)	Interferon-induced guanylate-binding protein 1	51,52
Omy100066751	326	GSONMT00080410001	Positive (0.84)	Tumor necrosis factor, alpha-induced protein 2 (tnfaip2)	9
Omy100128008	1,232	GSONMT00070499001	Positive (0.82)	Complement protein component C7-1 (c7-1)	66
Omy100063056	1,263	GSONMT00075049001	Positive (0.85)	Tumor necrosis factor, alpha-induced protein 3 (tnfaip3)	9
Omy200053140	1,510	GSONMT00071335001	Negative (0.84)	NF-kappa-B inhibitor alpha	9

References are provided for some of the protein-coding genes with previously described functions in immunity or association with microbial infection/pathogenesis.
