# Supplementary material for: Differential expression of long non-coding RNAs in three genetic lines of rainbow trout in response to infection with Flavobacterium psychrophilum
Source: Sci Rep. 2016 Oct 27;6:36032. doi: 10.1038/srep36032 (PMC5081542; doi:10.1038/srep36032)
Supplement: Supplementary Dataset 1 [file srep36032-s1.doc]

**Differential expression of long non-coding RNAs in three genetic lines of rainbow trout in response to infection with *Flavobacterium psychrophilum***

Bam Paneru^1^, Rafet Al Tobasei^2^, Yniv Palti^3^, Gregory D. Wiens^3^ and Mohamed Salem^1,2*^

^1^Department of Biology and Molecular Biosciences Program, Middle Tennessee State University, Murfreesboro, TN, 37132, U.S.

^2^Computational Science Program, Middle Tennessee State University, Murfreesboro, TN 37132, U.S.

^3^The National Center for Cool and Cold Water Aquaculture, USDA Agricultural Research Service, Kearneysville, WV 25430, U.S.

*Correspondence:

Mohamed Salem

Department of Biology and Molecular Biosciences Program,

Middle Tennessee State University,

Murfreesboro, TN, 37132, U.S.

Mohamed.salem@mtsu.edu

**Supplementary Dataset 1A.**: Summary statistics of 24 RNA seq libraries with different genetic lines, time, infection status and tank replicate

| **Genetic line** | **Day** | **Infection, Tank** | **Biosample Accession No** | **Total reads** | **Mapped reads** | **Percentage reads mapped (%)** | **Uniquely mapped reads** | **Total number of expressed lncRNAs (RPKM ≥5.0)** | **Total number of expressed lncRNAs (RPKM ≥1.0)** | **Total number of expressed lncRNAs (RPKM ≥0.50)** | **% of expressed genes (RPKM ≥0.50)** |
| --- | --- | --- | --- | --- | --- | --- | --- | --- | --- | --- | --- |
| ARS-Fp-R | 1 | Fp, Tk25 | SAMN03014722 | 20,061,852 | 1,761,205 | 8.8 | 1,663,742 | 19,540 | 26,371 | 27,561 | 88.4 |
|  |  | Fp, Tk26 | SAMN03014723 | 20,226,280 | 1,672,737 | 8.3 | 1,580,463 | 17,868 | 24,824 | 26,156 | 83.8 |
|  | 5 | Fp, Tk25 | SAMN03014726 | 21,409,329 | 1,769,707 | 8.3 | 1,672,380 | 18,718 | 25,562 | 26,834 | 86 |
|  |  | Fp, Tk26 | SAMN03014727 | 23,958,681 | 2,038,391 | 8.5 | 1,929,750 | 19,728 | 26,518 | 27,885 | 89.4 |
|  | 1 | PBS, Tk27 | SAMN03014724 | 22,129,914 | 1,795,272 | 8.1 | 1,697,092 | 19,482 | 26,241 | 27,491 | 88.1 |
|  |  | PBS, Tk28 | SAMN03014725 | 23,909,110 | 1,904,430 | 8 | 1,801,598 | 19,978 | 26,747 | 28,018 | 89.8 |
|  | 5 | PBS, Tk27 | SAMN03014728 | 24,361,298 | 2,009,877 | 8.3 | 1,899,213 | 19,311 | 26,127 | 27,559 | 88.3 |
|  |  | PBS, Tk28 | SAMN03014729 | 23,318,224 | 1,917,703 | 8.2 | 1,809,859 | 19,399 | 26,284 | 27,636 | 88.6 |
| ARS-Fp-C | 1 | Fp, Tk33 | SAMN03014738 | 20,940,097 | 1,734,138 | 8.3 | 1,642,815 | 20,027 | 26,680 | 27,806 | 89.1 |
|  |  | Fp, Tk34 | SAMN03014739 | 19,151,755 | 1,534,009 | 8 | 1,450,878 | 18,174 | 25,184 | 26,340 | 84.4 |
|  | 5 | Fp, Tk33 | SAMN03014742 | 21,117,398 | 1,713,769 | 8.1 | 1,618,904 | 18,796 | 25,550 | 26,767 | 85.8 |
|  |  | Fp, Tk34 | SAMN03014743 | 21,763,498 | 1,800,967 | 8.3 | 1,699,043 | 19,159 | 26,005 | 27,256 | 87.4 |
|  | 1 | PBS, Tk35 | SAMN03014740 | 20,314,994 | 1,655,407 | 8.2 | 1,567,306 | 17,357 | 24,396 | 25,710 | 82.4 |
|  |  | PBS, Tk36 | SAMN03014741 | 20,372,060 | 1,643,044 | 8.1 | 1,554,591 | 19,555 | 26,351 | 27,437 | 88 |
|  | 5 | PBS, Tk35 | SAMN03014744 | 24,480,995 | 2,043,423 | 8.4 | 1,929,929 | 18,728 | 25,650 | 27,153 | 87 |
|  |  | PBS, Tk36 | SAMN03014745 | 20,535,582 | 1,653,545 | 8.1 | 1,561,351 | 18,880 | 25,776 | 26,941 | 86.4 |
| ARS-Fp-S | 1 | Fp, Tk29 | SAMN03014730 | 17,389,164 | 1,392,049 | 8 | 1,317,455 | 19,201 | 25,958 | 26,878 | 86.2 |
|  |  | Fp, Tk30 | SAMN03014731 | 21,095,859 | 1,737,084 | 8.2 | 1,644,537 | 19,005 | 25,820 | 27,089 | 86.8 |
|  | 5 | Fp, Tk29 | SAMN03014734 | 19,446,430 | 1,556,366 | 8 | 1,473,769 | 19,378 | 26,118 | 27,172 | 87.1 |
|  |  | Fp, Tk30 | SAMN03014735 | 23,427,268 | 1,887,381 | 8.1 | 1,784,521 | 19,520 | 26,237 | 27,526 | 88.2 |
|  | 1 | PBS, Tk31 | SAMN03014732 | 20,622,290 | 1,631,915 | 7.9 | 1,545,298 | 18,861 | 25,810 | 26,975 | 86.5 |
|  |  | PBS, Tk32 | SAMN03014733 | 24,139,642 | 1,961,550 | 8.1 | 1,856,305 | 19,849 | 26,643 | 27,910 | 89.5 |
|  | 5 | PBS, Tk31 | SAMN03014736 | 22,371,928 | 1,820,511 | 8.1 | 1,718,566 | 19,576 | 26,350 | 27,607 | 88.5 |
|  |  | PBS, Tk32 | SAMN03014737 | 22,338,190 | 1,772,639 | 7.9 | 1,676,640 | 19,054 | 25,976 | 27,257 | 87.4 |
| Average |  |  |  | **21,620,076.60** | **1,766,963.30** | **8.2** | **1,670,666.90** | **19,131.00** | **25,965.80** | **27,206.80** | **87.2** |

**Supplementary Dataset 1B:** Real time PCR validation of randomly selected differentially expressed lncRNAs. All 7 lncRNAs were significantly different in RNA-Seq and real time PCR analysis (except Omy200194608). (FDR-corrected *p-*value < 0.05 for RNA-Seq and Mann Whitney U test *p*-value < 0.05 for real time PCR).


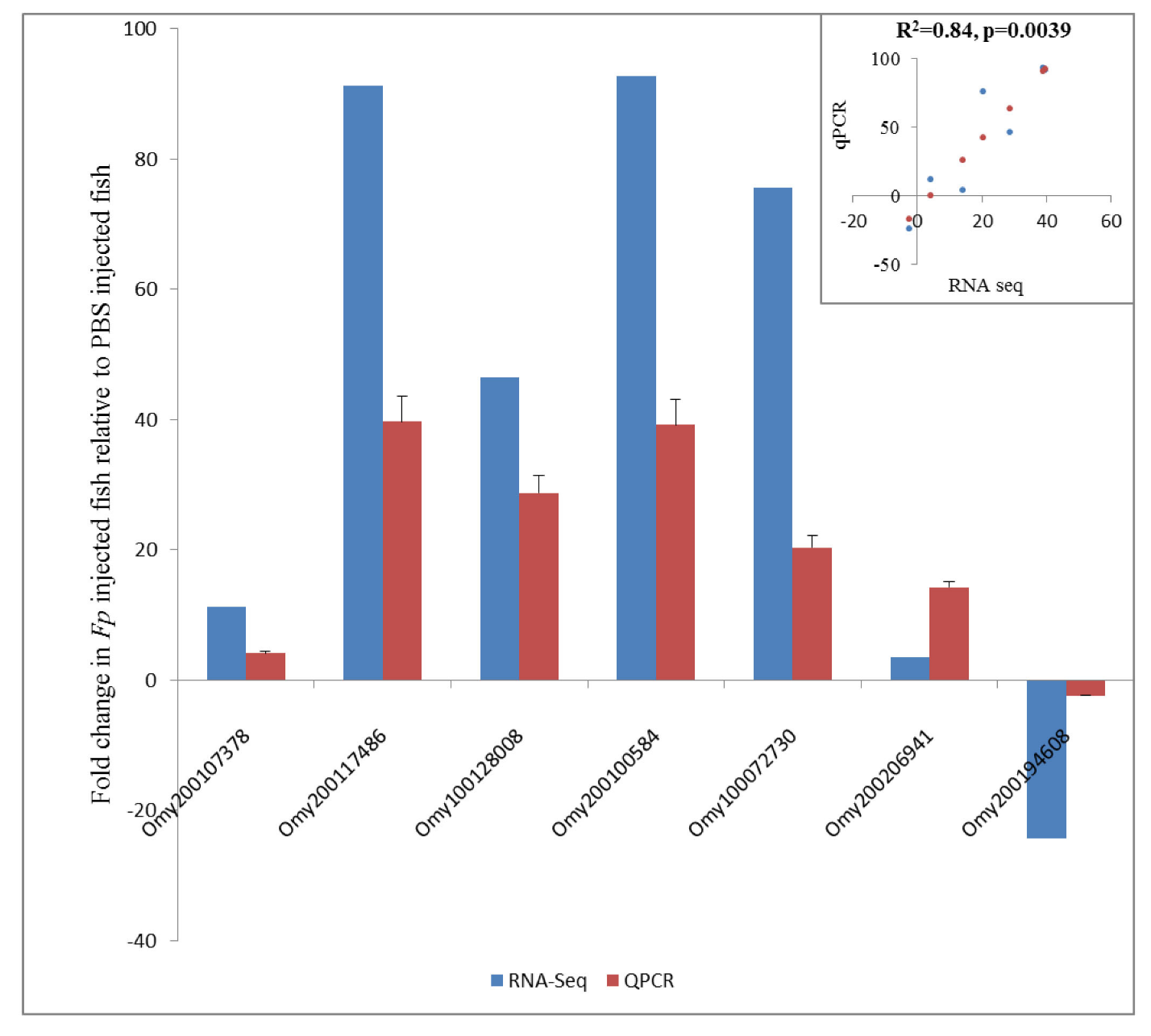


**Supplementary Dataset 1C:** Primer sequences and real time PCR cycling program conditions used for lncRNA expression validation

**Supplementary Dataset 1D:** Classification of DE lncRNA in response to *Fp* challenge based on their intersection with protein-coding genes and number of lncRNAs in each class.

**Supplementary Dataset 1E.** Strand specific PCR Method used in validation of strand orientation of some of lncRNAs transcripts relative to their protein coding loci counterparts.
